# Prevalence and distribution of ossification of the supra/interspinous ligaments in symptomatic patients with cervical ossification of the posterior longitudinal ligament of the spine: a CT-based multicenter cross-sectional study

**DOI:** 10.1186/s12891-016-1350-y

**Published:** 2016-12-01

**Authors:** Kanji Mori, Toshitaka Yoshii, Takashi Hirai, Akio Iwanami, Kazuhiro Takeuchi, Tsuyoshi Yamada, Shoji Seki, Takashi Tsuji, Kanehiro Fujiyoshi, Mitsuru Furukawa, Soraya Nishimura, Kanichiro Wada, Masao Koda, Takeo Furuya, Yukihiro Matsuyama, Tomohiko Hasegawa, Katsushi Takeshita, Atsushi Kimura, Masahiko Abematsu, Hirotaka Haro, Tetsuro Ohba, Masahiko Watanabe, Hiroyuki Katoh, Kei Watanabe, Hiroshi Ozawa, Haruo Kanno, Shiro Imagama, Zenya Ito, Shunsuke Fujibayashi, Masashi Yamazaki, Morio Matsumoto, Masaya Nakamura, Atsushi Okawa, Yoshiharu Kawaguchi

**Affiliations:** 1 Department of Orthopaedic Surgery, Shiga University of Medical Science, Tsukinowa-cho, Seta, Otsu, Shiga 520-2192 Japan; 2 Department of Orthopedic Surgery, Tokyo Medical and Dental University, 1-5-45 Yushima, Bunkyo-Ku, Tokyo, 113–8519 Japan; 3 Department of Orthopedic Surgery, School of Medicine, Keio University, 35 Shinanomachi, Shinjuku-Ku, Tokyo, 160–8582 Japan; 4 Department of Orthopedic Surgery, National Hospital Organization Okayama Medical Center, 1711–1Tamasu, Okayama, 701–1154 Japan; 5 Department of Orthopedic Surgery, Faculty of Medicine, University of Toyama, 2630 Sugitani, Toyama, 930–0194 Japan; 6 Department of Orthopedic Surgery, Kitasato Institute Hospital, 5-9-1, Shirokane, Minato-ku, Tokyo, 108-8642 Japan; 7 Department of Orthopedic Surgery, National Hospital Organization Murayama Medical Center, 2-37-1, Gakuen, Musashimurayama, Tokyo, 208-0011 Japan; 8 Department of Orthopedic Surgery, Keiyu Hospital, 3-7-3, Minatomirai, Nishi-Ku, Yokohama, Kanagawa 220-8521 Japan; 9 Department of Orthopedic Surgery, Hirosaki University Graduate School of Medicine, 53 Honcho, Hirosaki, Aomori, 036–8203 Japan; 10 Department of Orthopedic Surgery, Chiba University Graduate School of Medicine, 1-8-1 Inohana, Chuo Ward, Chiba, 260–0856 Japan; 11 Department of Orthopedic Surgery, Hamamatsu University School of Medicine, 1-20-1 Handayama, Hamamatsu, Shizuoka, 431–3125 Japan; 12 Department of Orthopedics, Jichi Medical University, 3311–1 Yakushiji, Shimotsuke, Tochigi, 329–0498 Japan; 13 Department of Orthopedic Surgery, Graduate School of Medicine and Dental Science, Kagoshima University, 8-35-1 Sakuragaoka, Kagoshima, 890–8520 Japan; 14 Department of Orthopedic Surgery, University of Yamanashi, 1110 Shimokato, Chuo Ward, Yamanashi, 409–3898 Japan; 15 Department of Orthopedic Surgery, Surgical Science, Tokai University School of Medicine, 143 Shimokasuya, Isehara, Kanagawa, 259–1143 Japan; 16 Department of Orthopedic Surgery, Niigata University Medicine and Dental General Hospital, 1–754 Asahimachidori, Chuo Ward, Niigata, 951–8520 Japan; 17 Department of Orthopaedic Surgery, Tohoku Medical and Pharmaceutical University, 1-12-1 Fukumuro Miyaginoku, Sendai, 983–8512 Japan; 18 Department of Orthopaedic Surgery, Tohoku University School of Medicine, 1–1 Seiryomachi, AobaWard, Sendai, Miyagi, 980–8574 Japan; 19 Department of Orthopedic Surgery, Nagoya University Graduate School of Medicine, 65 Tsurumaicho, Showa Ward, Nagoya, Aichi 466–0065 Japan; 20 Department of Orthopedic Surgery, Graduate School of Medicine, Kyoto University, 54 Kawaharacho, Shogoin, Sakyo-ku, Kyoto, 606–8507 Japan; 21 Department of Orthopedic Surgery, Faculty of Medicine, University of Tsukuba, 2-1-1 Amakubo, Tsukuba, Ibaraki 305–8576 Japan; 22 Japanese organization of the Study for Ossification of Spinal Ligament (JOSL), Otsu, Japan

**Keywords:** Supra/interspinous ligaments, Ossification, Ankylosed spine, Computed tomography, OPLL, OLF, DISH, Whole spine, Prevalence, Epidemiology

## Abstract

**Background:**

Supra/interspinous ligaments connect adjacent spinous processes and act as a stabilizer of the spine. As with other spinal ligaments, it can become ossified. However, few report have discussed ossification supra/interspinous ligaments (OSIL), so its epidemiology remains unknown. We therefore aimed to investigate the prevalence and distribution of OSIL in symptomatic patients with cervical ossification of the posterior longitudinal ligament (OPLL).

**Methods:**

The participants of our study were symptomatic patients with cervical OPLL who were diagnosed by standard radiographs of the cervical spine. The whole spine CT data as well as clinical parameters such as age and sex were obtained from 20 institutions belong to the Japanese Multicenter Research Organization for Ossification of the Spinal Ligament (JOSL). The prevalence and distribution of OSIL and the association between OSIL and clinical parameters were reviewed. The sum of the levels involved by OPLL (OP-index) and OSIL (OSI-index) as well as the prevalence of ossification of the nuchal ligament (ONL) were also investigated.

**Results:**

A total of 234 patients with a mean age of 65 years was recruited. The CT-based evidence of OSIL was noted in 68 (54 males and 14 females) patients (29%). The distribution of OSIL showed a significant thoracic preponderance. In OSIL-positive patients, single-level involvement was noted in 19 cases (28%), whereas 49 cases (72%) presented multi-level involvement. We found a significant positive correlation between the OP-index grade and OSI-index. ONL was noted at a significantly higher rate in OSIL-positive patients compared to negative patients.

**Conclusions:**

The prevalence of OSIL in symptomatic patients with cervical OPLL was 29%. The distribution of OSIL showed a significant thoracic preponderance.

## Background

The ossification of the posterior longitudinal ligament (OPLL) and ossification of the ligamentum flavum (OLF) are characterized by the replacement of the posterior longitudinal ligament and ligamentum flavum by ectopic new bone formation, respectively [1, 2]. The most frequent OPLL [3] and OLF [2] lesions are the cervical spine and the thoracic spine, respectively. OPLL often causes a narrow spinal canal and has been recognized as one of the causes of cervical myelopathy and/or radiculopathy [1, 3]. OLF is also well known as one of the causes of thoracic myelopathy through the compression of the spinal cord from the posterolateral side [2]. Some cases with ossification of the anterior longitudinal ligament (OALL) have been reported. Although OALL is not directly involved in the spinal canal, the longitudinal spread of OALL is known as diffuse idiopathic skeletal hyperostosis (DISH), which was reported by Resnick [4]. To date, DISH is a poorly understood systemic disease characterized by not only progressive OALL but also peripheral entheses [4, 5]. Ankylosed spine due to DISH yields biomechanical changes of the spinal system and acts as long bones, which can develop several manifestations peculiar to DISH [5–9]. Therefore, many clinicians pay attention to DISH in recent practice [5]. Theoretically, the continuous multi-level ossification of supra/interspinous ligaments (OSIL) may also cause the biomechanical changes of the spinal system and development of several manifestations similar to DISH. However, to date, few reports have discussed about OSIL. Although previous reports suggested that there is a pathophysiological association between DISH and ossification of other spinal ligaments such as OPLL [10, 11]. However, the characteristics in patients with OSIL are unknown. Thus, a multicenter study based on whole spine computed tomography (CT) data of symptomatic patients with cervical OPLL was conducted. The purpose of our study was to elucidate the prevalence and distribution of OSIL in symptomatic patients with cervical OPLL. We further investigated the association between OSIL and OPLL and ossification of the nuchal ligament (ONL) [12].

## Methods

Our study was conducted by Japanese Multicenter Research Organization for Ossification of the Spinal Ligament (JOSL), a specially commission instituted by the Japanese Ministry of Health, Labor and Welfare Health study. Written informed consent was obtained from each participant before registration at each institution. The local ethics committee of each institute approved this study.

### Participants

The participants of our study were symptomatic patients with cervical OPLL who were diagnosed by standard radiographs of the cervical spine. The definitive diagnosis of cervical OPLL on standard radiographs was determined as an ossification thicker than at least 3 mm within the posterior longitudinal ligament of the spine. If the OPLL was responsible for symptoms such as myelopathy, the cases with single level OPLL were also included. All standard radiographs were evaluated by an experienced spine surgeon at each participating institute. All enrolled patients had undertaken subsequent whole spine CT examination. The presence of OPLL in all eligible cases by standard radiograph was confirmed by subsequent CT. CT data as well as clinical parameters such as age, sex, presence of diabetes mellitus (DM) and body mass index (BMI) were obtained from 20 institutions belong to the JOSL. Patients who had a past history of anterior spinal decompression surgery for the treatment of OPLL and who were younger than 15 years were excluded from our study.

We calculated an adequate sample size in comparison of the OSIL-indexes among the 3 groups based on the OP-indexes. Because the OSIL-index was unknown before we conducted this study, we used a standard effect size: 0.25 in the power analysis for multiple comparisons. Then, we determined the minimum sample size to be 230 (α = 0.05, β = 0.9), considering the possibility of obtaining invalid data in 10% of the samples. Accordingly, a total of 234 patients (57 females and 177 males) with a mean age of 65 years (range, 33–93 years) were included in the analysis.

### Radiologic examination

All CT data were evaluated by five experienced spine surgeons (KM, TH, KT, AI and TY). Differences were settled by consensus to minimize intra- and inter-observer bias and errors. Before evaluation, the average Kappa coefficient of inter- and intra-observer agreement was determined by reviewing the same CT data of the 20 patients.

As there are no universally-approved criteria for OSIL; we counted the OSIL completely bridging adjacent spinous processes in our study. OSIL completely bridging at least four contiguous spinous processes were determined to be diffuse OSIL (DOSIL) according to the DISH criteria established by Resnick and Niwayama [4].

In addition, consistent with a previous report [13], the ossification index (OS-index) was determined. The OS-index is defined as the total number of vertebral body and intervertebral disc levels involved by ossification of the ligament. Therefore, the OS-indexes for OPLL and OSIL were represented as the OP-index and OSI-index, respectively. We subdivided the patients into three subgroups according to the cervical OP-index, which is consistent with a previous report [14]: Grade 1: patients with a cervical OP-index of 5 or less; Grade 2: patients with a cervical OP-index of 6–9; and Grade 3: patients with a cervical OP-index of 10 or more. The presence of ONL was also evaluated.

### Statistical analysis

Student’s unpaired *t*-test, chi-square test, Tukey post hoc test and Pearson’s product moment correlation coefficient were used when appropriate. *P* < 0.05 was considered statistically significant. The software application used for the analysis was SPSS for Windows version 22.0 (SPSS Institute, Chicago, IL).

## Results

The average Kappa coefficient of inter-observer agreement by reviewing the same images of 20 patients for OPLL, OSIL and ONL were 0.76, 0.79 and 1.0, respectively. The intra-observer agreement for OPLL, OSIL and ONL were 0.94, 0.94 and 1.0, respectively.

**Fig. 1:**
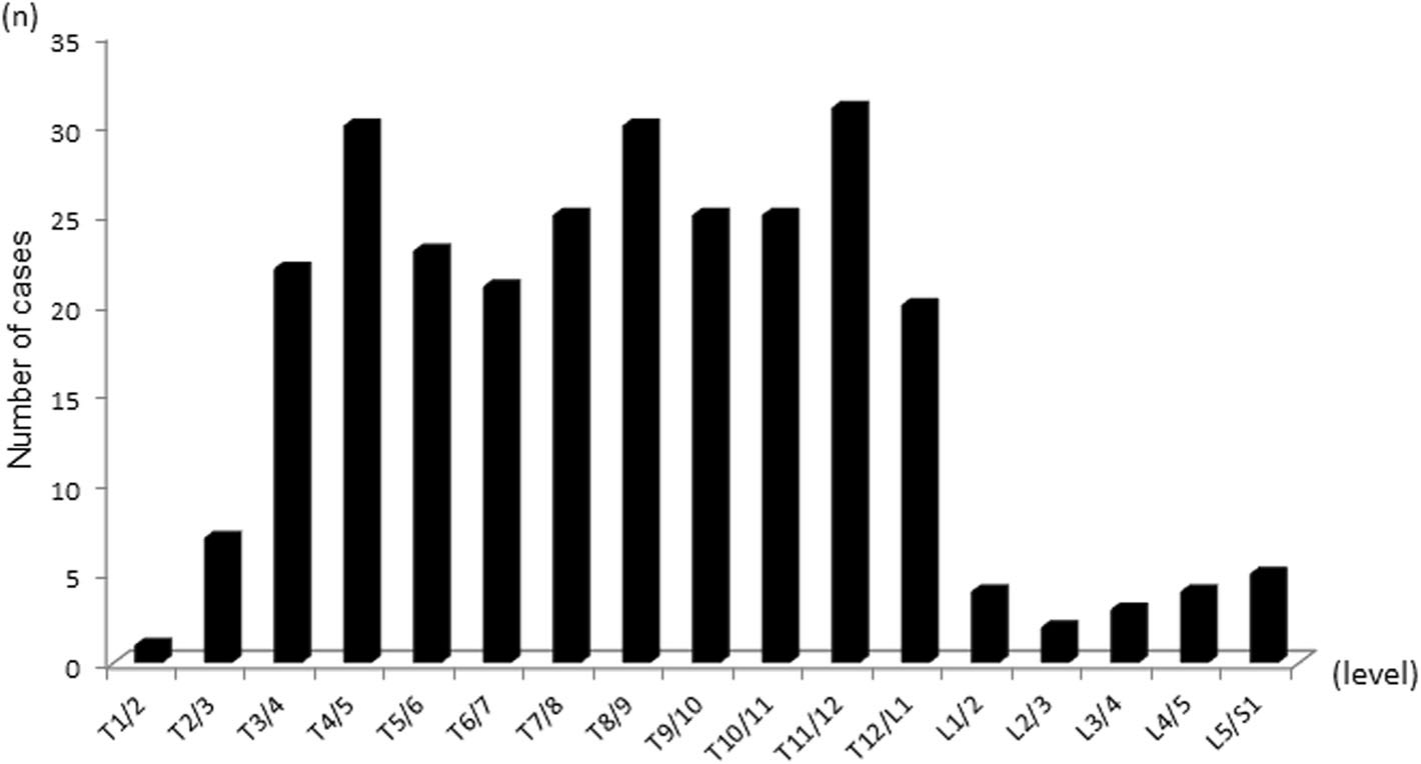
Distribution of OSIL in the thoracolumbar region of 234 symptomatic patients with cervical OPLL

**Fig. 2:**
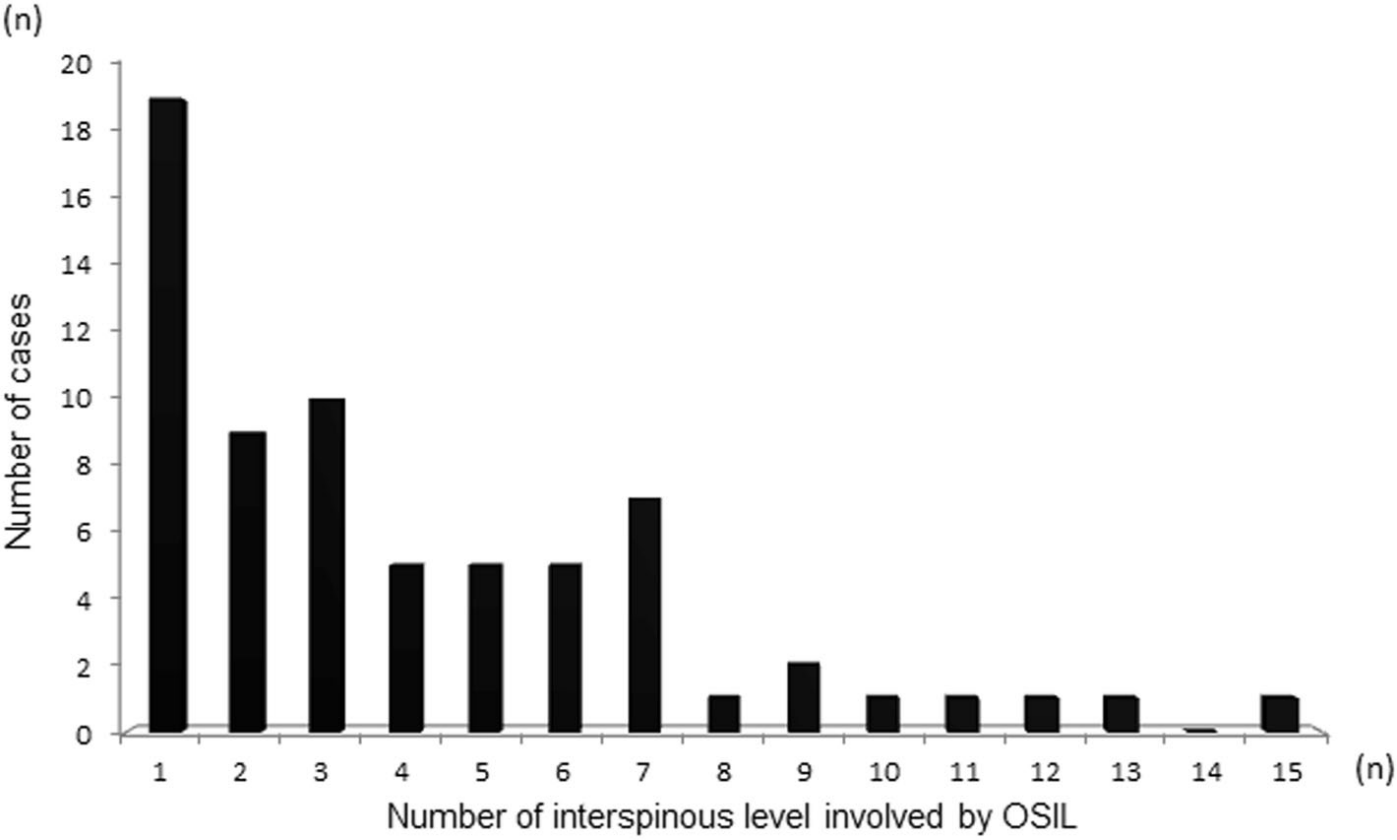
Distribution of the patients with OSIL according to the number of involved interspinous levels

Among 234 symptomatic patients with cervical OPLL, OSIL was noted in 68 patients (29%) (54 males and14 females). The mean age of OSIL-positive patients was significantly higher than that of negative patients (OSIL-positive: 70 years *vs.* OSIL-negative: 63 years, *p* < 0.01, Table 1). Figure 1 shows the distribution of OSIL in the thoracolumbar region. OSIL were noted for 278 interspinous levels. OSIL were noted at a higher rate in the thoracic region (260 interspinous levels) than in the lumbar region (18 interspinous levels). In OSIL-positive patients, single-level involvement was noted in 19 cases (28%), whereas 49 cases (72%) presented multi-level involvement. Among those with multi-level involvements, the highest number of involved level with OSIL reached 15 levels. These findings are shown in Fig. 2. The number of cases with multi-level involvements was small; we subdivided OSIL-positive patients into 2 subgroups according to DISH criteria [3]: DOSIL and non DOSIL for our analyses. DOSIL consists of 40% out of total OSIL cases (68).

**Table 1:** Characterization of the participants

	OSIL (+)	OSIL (-)	*p*-value	DOSIL (+)	DOSIL (-)	*p*-value
Sex (M/F)	54/14	123/43	0.39	21/6	156/51	0.78
Age (yr)	70 ± 9.7	63 ± 9.7	<0.01	71 ± 9.1	65 ± 11	<0.01
BMI (kg/m^2^)	25 ± 4.8	26 ± 4.8	0.55	26 ± 6.5	25 ± 4.6	0.83
DM (+) n (%)	21 (31)	50 (30)	0.91	11 (41)	60 (29)	0.21
OP-index	2.2 ± 0.73	1.7 ± 0.70	<0.01	2.3 ± 0.68	1.7 ± 0.74	<0.01
ONL (+) n (%)	46 (68)	85 (51)	0.021	21 (78)	110 (53)	0.015

*OSIL* ossification of supra/inter spinous ligaments, *DOSIL* diffuse OSIL, *M* male, *F* female, *BMI* body mass index, DM (+): presence of diabetes mellitus, OP-index: ossification index for ossification of the posterior longitudinal ligament of the spine, ONL (+): presence of ossification of the nuchal ligament

Age, BMI and OP-index were represented by mean ± standard deviation

**Fig. 3:**
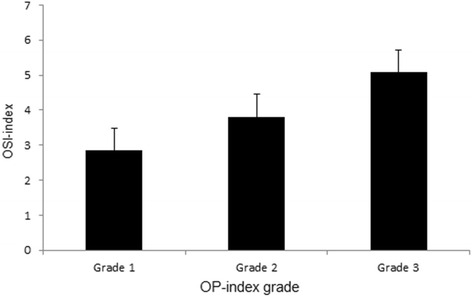
Sum of the levels involved by OSIL (OSI-index) according to the sum of the levels involved by OPLL (OP-index) grade

The mean OP-index in OSIL-positive patients was significantly higher than that of OSIL-negative patients: 2.2 and 1.7 for the OSIL-positive and negative patients, respectively (*p* < 0.01, Table 1). The same trend was found between DOSIL-positive and negative patients (*p* < 0.01, Table 1). We further investigated OSI-index according to the OP-index grade and also found a positive correlation between OP-index grade and OSI-index (*p* < 0.01, r = 0.32, Fig. 3). We found a significant difference in the OSI-index among the three OP-index grades (*p* < 0.01).

ONL were found in 46 out of 68 OSIL-positive patients (68%) and 21 out of 27 DOSIL-positive patients (78%). ONL were noted at a significantly higher rate in both OSIL-positive (*p* = 0.021) and DOSIL-positive patients (*p* = 0.015) compared to those of negative patients (Table 1).

We evaluated the association between sex, BMI and presence of DM and OSIL status, and no significant difference was found (Table 1).

## Discussion

To date, our study is the largest multicenter study to investigate whole spine CT images in symptomatic patients with cervical OPLL, including disclosing the CT-based prevalence and distribution of OSIL. The results of the average Kappa coefficient of inter- and intra-observer agreement by reviewing the same images of 20 patients indicated an excellent and substantial agreement [15]. We believe that the present data were reliable.

The supraspinous ligament connects the tips of the spinous processes from the 7th cervical vertebra to the sacrum [16]. The interspinous ligaments are thin and membranous ligaments that connect adjacent spinous processes of the vertebra in the spine [16]. These ligaments act as a stabilizer of the spine [16]. The thoracic spine has a limited range of motion due to its characteristic anatomy compared to that of the cervical and lumbar spine. The reason for a predominantly thoracic distribution of OSIL remains unknown; however it is likely that the spinal range of motion may have some impact on the distribution of OSIL.

Accumulating results show that a consecutively ankylosed spine due to DISH yields biomechanical changes in the spinal system and develops several characteristic manifestations including spinal fractures resembling those of long bone [5–9]. It can also develop a significant displacement of the fracture site even though it was no or minimal displacement after a relatively minor trauma [5], which may cause both patients’ and/or doctors’ delay [6–9]. Fractures in patients with ankylosed spine often resulted from minor trauma such as falls from a standing/sitting level, which can cause an underestimation of injury severity by both patients and doctors [8, 9]. In addition, pre-existing pathologic changes of the spine prevent correct diagnosis. Consequently, some patients experience a sudden neurological deterioration [8, 9]. Secondary neurological deterioration may also occur by inadequate immobilization, careless transfers or imprudent manipulation of these patients [8, 9]. Moreover, the patients with an ankylosed spine can develop several complications such as aspiration pneumoniae, kyphotic deformity, myelopathy, and unexpected hyperextension fracture-dislocation of the thoracic spine with paraplegia by position during retroperitoneal surgery [17–19]. A systematic review of the literature revealed that the complication rate and overall mortality within 3 months after injury in DISH patients were 32.7% and 20.0%, respectively [9]. These findings suggested that we must pay attention to the presence of ankylosed spine for the spine care [5, 8, 9].

Consecutively ankylosed spine by means of OSIL completely bridging adjacent spinous processes may cause biomechanical changes of the spinal system, which is similar to DISH. Nevertheless, to the best of our knowledge, OSIL have received less attention to date. The reason remains unknown; however it is likely that OSIL does not directly develop the neurological compromise that is observed in OPLL and OLF. In addition, in contrast with DISH, diagnosis of OSIL by standard radiographs is difficult.

The nuchal ligament is the equivalent structure of the supraspinous ligament in the cervical spine [16]. As a localized ossification, ONL were noted to be significantly more frequent in OSIL/DOSIL-positive patients compared to those of negative patients. The ONL-positive rate was higher in DOSIL-positive patients than in OSIL-positive patients. In addition, the same trend was found between the severity of the OSIL and OP-index (Table 1). Furthermore, interpreted from a cervical OPLL viewpoint, we found a positive correlation between the cervical OP-index grade and the OSI-index (Fig. 3). Thus, these findings suggested a positive association between OSIL and OPLL, and it is possible that we can use the cervical OP-index grade as an indicator of ossification of the general spinal ligaments.

Alternatively, no significant difference was found between sex, BMI and presence of DM and OSIL/DOSIL status. These findings may be attributed to the character of the participants in our study. That is, the participants in our study were exclusively patients with cervical OPLL, which are significantly predominant in males with high BMI and DM involvement [3]. This background can mask the association between these factors and OSIL/DOSIL status.

Our study has several limitations. First, our study is not a population-based study, and thus true prevalence and distribution of OSIL cannot be determined. Our findings are those of symptomatic cervical OPLL patients only and cannot be generalized to the general population. Although CT allows more precise evaluation of the ossification of spinal ligaments compared to radiography [20, 21], much higher radiation dose of CT makes it ethically impossible to perform an observational epidemiological population study using CT scan. In addition, we [13, 20, 21] and others [22] have reported that patients with symptomatic cervical OPLL frequently had tandem ossifications at the thoracolumbar region, which sometimes required additional surgeries. It is therefore important to evaluate the whole spine so as not to overlook the latent risk of tandem ossifications in the thoracolumbar region when we treat patients with cervical OPLL [13, 20–22]. As another limitation, it is possible that cases with small cervical OPLL were neglected. All eligible cases by standard radiographs were OPLL positive by subsequent CT scan; we therefore believe that there were no false-positive cases. Another limitation is that, due to the nature of retrospective studies, we cannot evaluate whether clinical manifestations such as stiffness, impaired mobility, symptomatic duration and other co-morbidity like medications are significantly associated with OSIL. Furthermore, the real impact of biomechanical changes to the spinal system due to OSIL on clinical manifestations remains unknown and should be determined in future studies. Despite these limitations, one favorable aspect of our study is that it is the largest multicenter study to investigate whole spine CT images in symptomatic patients with cervical OPLL and is the first to elucidate the prevalence and distribution of OSIL.

## Conclusions

The CT-based prevalence of OSIL in symptomatic patients with cervical OPLL was 29%, and its distribution showed a significant thoracic preponderance. Among OSIL-positive patients, 40% were classified as DOSIL. Awareness of the presence of consecutively ankylosed spine may be very important for the spine care.

## Abbreviations

BMI: Body mass index
CT: Computed tomography
DISH: Diffuse idiopathic skeletal hyperostosis
DM: Diabetes mellitus
DOSIL: Diffuse ossification of supra/interspinous ligaments
JOSL: Japanese Multicenter Research Organization for Ossification of the Spinal Ligament
OALL: Ossification of the anterior longitudinal ligament
OLF: Ossification of the ligamentum flavum
ONL: Ossification of the nuchal ligament
OP-index: OS-index for OPLL
OPLL: Ossification of the posterior longitudinal ligament
OSI-index: OS-index for OSIL
OSIL: Ossification of supra/interspinous ligaments
OS-index: Ossification index
